# Computer-Assisted Planning for Stereoelectroencephalography (SEEG)

**DOI:** 10.1007/s13311-019-00774-9

**Published:** 2019-08-20

**Authors:** Vejay N. Vakharia, Rachel Sparks, Anna Miserocchi, Sjoerd B. Vos, Aidan O’Keeffe, Roman Rodionov, Andrew W. McEvoy, Sebastien Ourselin, John S. Duncan

**Affiliations:** 1 Department of Clinical and Experimental Epilepsy, Institute of Neurology, University College London, London, UK; 2 National Hospital for Neurology and Neurosurgery, Queen Square, London, UK; 3 Chalfont Centre for Epilepsy, Chalfont St Peter, UK; 4 School of Biomedical Engineering and Imaging Sciences, St Thomas’ Hospital, King’s College London, London, UK; 5 Wellcome Trust EPSRC Interventional and Surgical Sciences, University College London, London, UK; 6 Department of Statistical Science, University College London, London, UK

**Keywords:** SEEG, Clinical decision support software, Epilepsy, Computer-assisted planning, EpiNav

## Abstract

Stereoelectroencephalography (SEEG) is a diagnostic procedure in which multiple electrodes are stereotactically implanted within predefined areas of the brain to identify the seizure onset zone, which needs to be removed to achieve remission of focal epilepsy. Computer-assisted planning (CAP) has been shown to improve trajectory safety metrics and generate clinically feasible trajectories in a fraction of the time needed for manual planning. We report a prospective validation study of the use of EpiNav (UCL, London, UK) as a clinical decision support software for SEEG. Thirteen consecutive patients (125 electrodes) undergoing SEEG were prospectively recruited. EpiNav was used to generate 3D models of critical structures (including vasculature) and other important regions of interest. Manual planning utilizing the same 3D models was performed in advance of CAP. CAP was subsequently employed to automatically generate a plan for each patient. The treating neurosurgeon was able to modify CAP generated plans based on their preference. The plan with the lowest risk score metric was stereotactically implanted. In all cases (13/13), the final CAP generated plan returned a lower mean risk score and was stereotactically implanted. No complication or adverse event occurred. CAP trajectories were generated in 30% of the time with significantly lower risk scores compared to manually generated. EpiNav has successfully been integrated as a clinical decision support software (CDSS) into the clinical pathway for SEEG implantations at our institution. To our knowledge, this is the first prospective study of a complex CDSS in stereotactic neurosurgery and provides the highest level of evidence to date.

**Electronic supplementary material:**

The online version of this article (10.1007/s13311-019-00774-9) contains supplementary material, which is available to authorized users.

## Introduction

Surgery can result in sustained seizure freedom in patients with drug-resistant focal epilepsy if the seizure onset zone (SOZ) can be resected [1]. Invasive EEG recordings are needed to identify the SOZ when noninvasive presurgical investigations are discordant, when a tailored resection is required and for mapping adjacent eloquent cortex [2, 3]. Over the last two decades, there has been a significant shift toward stereoelectroencephalography (SEEG) from subdural grid placement in most epilepsy surgery centers [4]. SEEG is a procedure in which electrodes are stereotactically inserted into 10–16 predefined brain regions and affords a comparatively favorable safety profile [5, 6] with rapid patient recovery time. Trajectory planning follows the formulation of an intracranial EEG sampling strategy, derived from consideration of seizure semiology, scalp EEG, and imaging data. Precise SEEG trajectory planning requires a number of parameters to be optimized, including accurate targeting of the anatomical structures of interest through an avascular corridor, drilling angle to the skull, intracerebral length, gray matter (GM) sampling, and avoidance of other SEEG electrodes. Planning is, therefore, a time-consuming process that requires multidisciplinary input. The risk of morbidity from SEEG in a recent meta-analysis was 1 per 287 electrodes which equates to 1 in every 29 patients implanted [7]. The greatest risk associated with SEEG is hemorrhage and it is imperative that all possible measures to mitigate this are employed.

Computer-assisted planning (CAP) enables parameters, which are thought to be most useful during preoperative planning, to be optimized in a systematic and time-saving manner. Such software has been classified by the FDA as “clinical decision support software” (CDSS) and legislation differentiates this from medical devices [8]. A working definition of CDSS is a system that “provides clinicians or patients with computer-generated clinical knowledge and patient-related information, intelligently filtered or presented at appropriate times, to enhance patient care” [9]. To be classified as a CDSS the software must: 1) be intended to display, analyze, or print medical information about a patient or 2) be intended to support or provide recommendations to a health care professional about prevention, diagnosis, or treatment of a disease but 3) not be intended to acquire, process, or analyze medical images or signals and 4) the healthcare professional must be able to review the basis for such recommendations [8]. Most current CDSSs provide clinicians with alerts or reminders, such as drug allergy status and are embedded within hospital electronic systems. More sophisticated CDSSs include disease-related scoring systems or utilize artificial intelligence to aid diagnosis or management.

EpiNav is a CDSS that is able to automatically generate multitrajectory SEEG plans in a fraction of the time required for manual planning. Previous studies of SEEG CDSSs have been retrospective comparisons with previously implanted manually planned trajectories [10–13]. These showed reduced risk scores with the use of CAP. To assess the real-world clinical utility of the automated trajectories, external blinded expert neurosurgeons rated the feasibility of both manual and automatically planned trajectories based on whether they would implant an electrode along the trajectory based on their individual practice. This revealed no difference between the acceptability of manual and CAP trajectories. Ratings for implanted manually planned trajectories were ~ 70%, highlighting the variability in surgical practice [13].

We report a prospective comparative study between CAP and manually planned SEEG trajectories in which the plan with the lowest mean risk score was stereotactically implanted.

## Methods

### Patient Demographics

Thirteen consecutive patients (7 male) with drug-resistant focal epilepsy undergoing SEEG as part of their routine clinical care at The National Hospital for Neurology and Neurosurgery, London, UK, were enrolled between July 2017 and January 2018. This study was granted by the National Research Ethics Service Committee London, approval reference: 12/LO/0377. Written consent was obtained from all patients prior to inclusion in the study. Patient age at the time of SEEG implantation was 33.5 ± 6.5 years (mean ± S.D.). Target regions for SEEG sampling were determined following a multidisciplinary team meeting in which the clinical history, semiology, video telemetry, imaging, neuropsychological, and neuropsychiatric assessments were reviewed (see Table 1). Following this, the EpiNav software was then utilized to assist the surgeon with the precise planning of the electrode trajectories, after the acquisition of vascular imaging.

**Table 1:** Patient demographics

No.	Age (years)	Onset of epilepsy (years)	Hemispheric language dominance (fMRI)	Semiology	Scalp EEG (contact names based on EEG 33 system)	Neuroimaging findings	Primary hypothesis of EZ
1	42	3	Left	1. Psychic aura 2. Automotor seizure 3. Dystonic posturing of the left arm 4. Post-ictal nose wiping with the right hand	Interictal: Intermittent right temporal slowing and right temporal sharp waves Ictal: Fast activity in posterior parietal region	1. Right mesial temporal sclerosis Hippocampal volumes: Right 2.34 cm^3^ Left 2.94 cm^3^ Ratio 79% 2. FDG PET inconclusive	Neocortical posterior quadrant onset with early temporal involvement
2	43	14	Left	1. Psychic aura 2. Complex motor/hyperkinetic seizure 3. Loss of awareness 4. Post-ictal speech difficulties	Interictal: Intermittent slow left and right temporal regions. Sharp waves left anterior temporal (F7 > LSPh > F11 > F3, ~ 50%) and right fronto-temporal (F8 > RSPh > F12 > T4, ~ 50%) Ictal: Left temporal discharges at onset of seizure	1. Normal structural imaging 2. FDG PET left frontal and temporal hypometabolism 3. Ictal SPECT inconclusive	Left frontal (neuropsychological testing implicates the dominant fronto-temporal region)
3	26	6	Left	1. Autonomic aura 2. Automotor seizure—vomiting followed by outstretched arms and clenched fists 3. Dialeptic—behavioral arrest with loss of awareness followed by oral automatisms 4. Secondary generalized tonic/clonic seizure	Interical: Polyspikes and sharp waves (F8 > FC6 > F4) in runs without clinical change Ictal: Right inferior frontal > right fronto-central onset with prominent ictal tachycardia	1. Normal structural imaging 2. FDG PET—mildly reduced tracer activity in both temporal lobes	Right hemispheric / insula (neuropsychological testing implicates the nondominant temporal region)
4	34	1	Left	1. Psychic/autonomic aura 2. Hyperkinetic seizure (bicycling movements in both legs)	Interictal: No abnormalities Ictal: Left temporal lobe onset	1. Left temporal hippocampal sclerosis 2. FDG PET—reduced metabolic activity in the left temporal lobe	Left fronto-temporal (neuropsychological testing unable to distinguish left frontal from temporal dysfunction due to language barrier) Possible nonepileptic attacks
5	25	9	Not performed	1. Unspecified aura 2. Dialeptic seizure 3. Left arm and leg tonic seizure 4. Axial tonic seizure	Interictal: Intermittent slow over vertex (Cz) and right fronto-central (F4 and C4) Ictal: Right fronto-central onset	1. Hemosiderin staining in the left superior frontal gyrus suggestive of cavernoma 2. FDG PET—minimal hypometabolism in left superior frontal region 3. Ictal SPECT—hyperperfusion in the left superior frontal and right frontal regions	Right mesial frontal lobe (neuropsychological testing suggests frontal lobe dysfunction)
6	37	7	Bilateral	1. Unspecified aura 2. Asymmetric tonic seizure (left arm extended) 3. Dialeptic seizure	Interictal: Very rare sharp waves anterior frontal region Ictal: Rhythmic activity (3–5 Hz) fronto-central region	1. Normal structural imaging 2. FDG PET—subtle reduction in metabolic activity in right frontal lobe 3. Ictal SPECT—hyperperfusion in the right frontal lobe	Right fronto-central (neuropsychological testing suggests dominant hemisphere dysfunction)
7	31	3	Right	1. Right arm somatosensory aura 2. Asymmetric tonic seizure (right arm) 3. Post-ictal right arm weakness 4. Dialeptic/automotor seizure	Interictal: Sharp waves left fronto-temporal (max F7/T7) with polyspikes and left posterior temporo-parietal (max P7) Ictal: Attenuation and low amplitude fast in left temporo-parietal region. Repetitive left temporo-parietal sharp waves	1. Extensive damage to left hemisphere lined by gliotic rim involving temporal, parietal, and insula lobes	Left centro-parietal (neuropsychological testing suggests widespread cerebral dysfunction maximally implicating the nondominant fronto-parietal region)
8	36	4	Left	1. Dialeptic seizure 2. Automotor seizure 3. Secondary generalized tonic/clonic seizure	Interictal: Sharp waves right temporal (max T4) 80% and sharp waves left anterior temporal (max F7) 20% Ictal: Onset nonlocalizable. Evolution more prominent over right hemisphere	1. Normal structural imaging 2. FDG PET—reduced metabolic activity in temporal lobes bilaterally, right more than left	Right fronto-temporal (neuropsychological testing did not provide any consistent lateralizing or localizing signs)
9	33	7	Left	1. Left arm and leg somatosensory aura 2. Autonomic aura 3. Automotor seizure 4. Secondary generalized tonic/clonic seizure	Interictal: Intermittent right temporal slowing Ictal: Right temporal onset with wider right hemispheric onset recorded in some seizures	1. Right hippocampal sclerosis Hippocampal volumes: Right 2.08 cm^3^ Left 2.53 cm^3^ Ratio 82%	Right temporal plus (neuropsychological testing suggests dominant fronto-temporal dysfunction)
10	19	15	Left	1. Bilateral visual/auditory aura 2. Automotor seizure 3. Secondary generalized tonic/clonic seizure	Interictal: Sharp wave left temporal (LSph and T7) and left frontal (Fp1 > F3 > Fz) Ictal: Spike and slow waves left hemisphere, maximal fronto-centro-temporal region with spread to the right	1. Normal structural imaging 2. FDG PET—mild reduction in metabolic activity in the left temporal lobe	Left fronto-temporal (neuropsychological testing suggests left temporal lobe dysfunction)
11	29	22	Left	1. Psychic aura 2. Automotor seizure—left hand and oral automatisms 3. Secondary generalized tonic/clonic seizure	Interictal: Sharp waves right temporal maximal (F12 and T8). Rare left temporal sharp waves (F11) Ictal: Regional right inferior temporal onset which evolves to rhythmic theta and propagation to the right parasagittal and left temporal regions	1. Right hippocampal sclerosis Hippocampal volumes: Right 2.14 cm^3^ Left 2.76 cm^3^ Ratio 77.6% 2. FDG PET – Reduced metabolic activity in the right temporal lobe	Right fronto-temporal (neuropsychological testing suggests right frontal lobe dysfunction)
12	32	22	Right	1. Bilateral somatosensory aura 2. Asymmetric tonic seizure—head turn to left with bilateral arm extension 3. Secondary generalized tonic/clonic seizure	Interictal: Sharp waves left fronto-central region (F3/FC1 > FC5) Ictal: Left fronto-central onset	1. Subtle left hippocampal sclerosis Hippocampal volumes: Right 3.02 cm^3^ Left 2.93 cm^3^ Ratio 97% 2. FDG PET—reduced hypometabolism over the left hemisphere most prominent in the left inferior frontal region	Left fronto-central (neuropsychological testing suggests dominant temporal lobe dysfunction)
13	36	27	Left	1. Dialeptic seizure 2. Automotor seizure 3. Secondary generalized tonic/clonic seizure	Interictal: Sharp waves right anterior temporal (50%), left anterior temporal (30%), right frontopolar (10%), and left frontopolar (5%) Right fronto-central paroxysmal fast activity Ictal: Right hemispheric activity at onset, maximal centro-parietal regions followed by temporal spread	1. Widening of the sulci over the right cerebral hemisphere suggestive of a perinatal right hemispheric insult 2. FDG PET—Reduced metabolic activity in right frontal and temporal lobes	Right fronto-temporal (neuropsychological testing did not provide any consistent lateralizing or localizing signs)

### Trajectory Planning

Manual planning was undertaken by experienced neurosurgeons in all patients with 3D models of the cortex and vascular segmentation prior to CAP (see below for description). Entry points on the scalp surface and target points within the structure of interest were manually determined and iterated to achieve a satisfactory solution that was labelled *plan 1*.

**Fig. 1:**
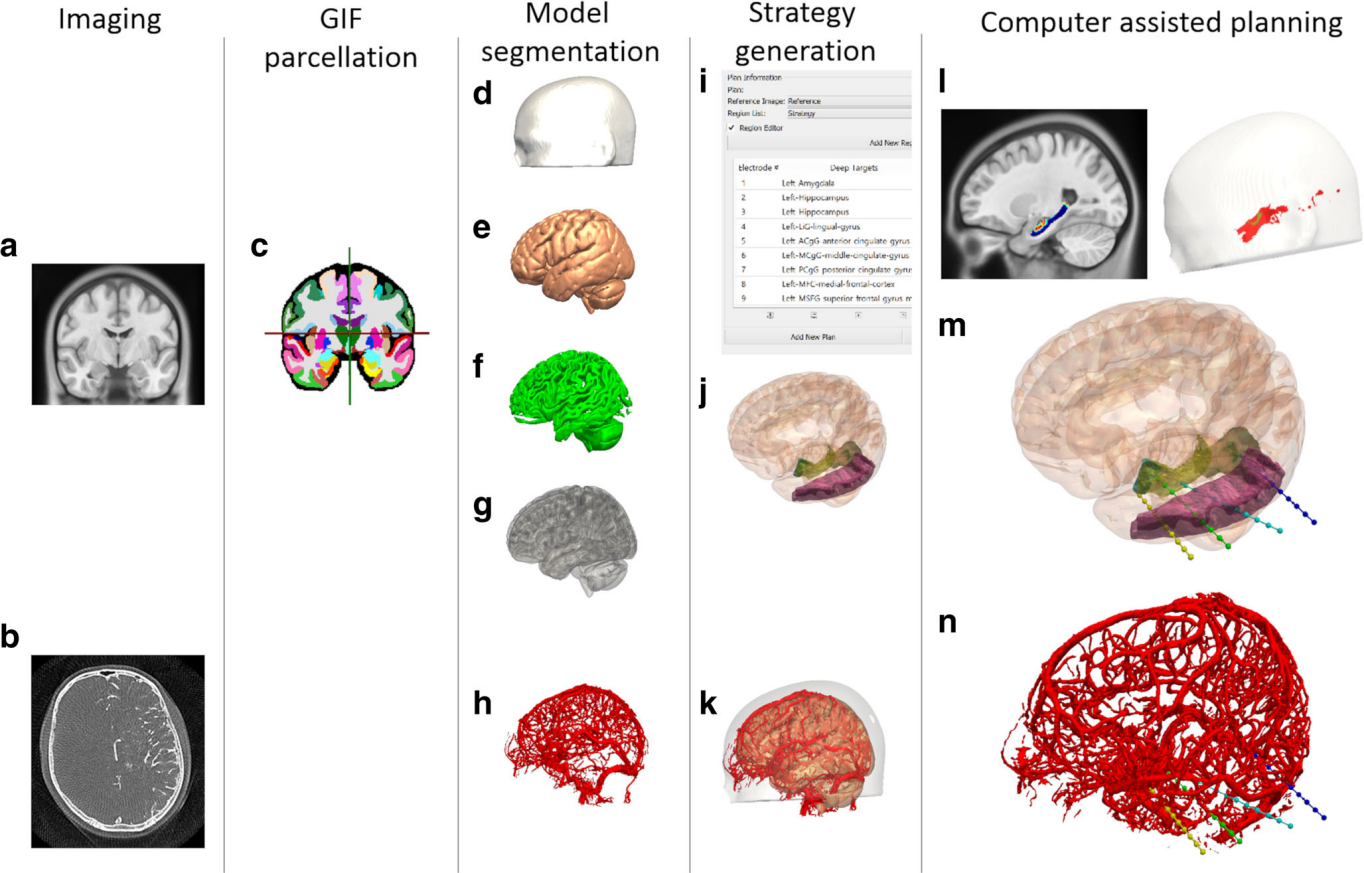
CAP image processing pipeline: imaging modalities required for CAP include a reference image (A), preferably a gadolinium-enhanced T1 image, and a vascular imaging modality (B). A whole brain parcellation (C) is generated from the T1 image. A model of the scalp (D) is generated from the reference image while models of the cortex (E), sulci (F), and gray matter (G) are automatically extracted. Vascular models (H) are derived from the vascular imaging following filter application and mesh cleaning. The implantation schema entry and target points are then selected from the whole brain parcellation (I) and brain ROIs are automatically segmented (J). In this case, amygdala, hippocampus, and lingual gyrus target regions are shown with the middle temporal gyrus as the entry region. A composite image of the scalp, brain, and vasculature is shown (K). Trajectories that exceed length, angle, and critical structure restrictions are removed from consideration. Risk maps for the target structure (only hippocampus shown) and corresponding entry zones are generated (L). CAP trajectories with shortest intracerebral length, orthogonal drilling angles, maximal gray matter sampling, and lowest trajectory risk score are provided (M). Generated trajectories also shown with vascular model (N). ROI = region of interest. Note: for clarity only temporal electrodes are shown

CAP was undertaken using EpiNav (UCL, London, UK). EpiNav is a multimodal imaging platform that allows manual as well as advanced multitrajectory automated planning [14], invasive EEG grid/electrode contact localization [15], SEEG signal visualization, source localization, and resection planning [16]. CAP was performed with a gadolinium-enhanced T1 MRI reference image and vascular segmentation (see Fig. 1). Patients also underwent digital subtraction catheter angiography (DSCA) with an intra-arterial contrast injection of the ipsilateral internal carotid artery and/or vertebral artery depending on the implantation strategy. A vessel extraction filter [17] was applied to the raw bone subtracted DSCA images prior to manual threshold setting, 3D model generation and mesh cleaning. A rigid registration of the bone-inclusive DSCA image to the reference image was then performed. A visual check of the vessel segmentation suitability and registration accuracy was performed before commencing planning. Whole brain parcellations and pseudo-CT images were generated from T1 MPRAGE sequences with a field-of-view (FOV) of 224 × 256 × 256 mm (antero-posterior, left-right, inferior-superior) and acquisition matrix of 224 × 256 × 256 for a voxel size of 1 mm isotropic (TE/TR/TI = 3.1/7.4/400 ms; flip angle 11°; parallel imaging acceleration factor 2) using geodesic information flows (GIF) [18]. Patient-specific 3D models of the cortex, sulci, GM, scalp, and SEEG entry and target regions were then generated from the GIF parcellations. The same models were utilized during manual planning as were required for CAP to ensure parity between the two planning methods. For a detailed description of the CAP algorithm, see Sparks et al. [12]. In brief, the DSCA vascular segmentation was used as a critical structure and the algorithm plans trajectories to remain as far from the vessel as possible, up to a distance of 1 cm. A minimum vessel distance of 3 mm was set based on previous accuracy data [19], whereby only 1% of implanted electrodes would exceed this distance from the planned trajectory. A risk score [12, 20], based on the cumulative distance of the planned trajectory from the vessel segmentation, was calculated using the following equation:

$$ R=\left\{\begin{array}{c}\sum \limits_i^N\frac{10-\mathrm{Dist}(i)}{N\left(10-3\right)},\kern0.75em \mathrm{Dist}(i)>3\\ {}1+\sum \limits_i^N\frac{3-\mathrm{Dist}(i)}{3N},\kern0.75em \mathrm{Dist}(i)\le 3\end{array}\right. $$

where *N* = 128, the total number of sampling nodes along the length of the trajectory and *i* denotes the indices of the individual node being computed. The risk score (*R*) is expressed as a value between 0 and 2, with values > 1 indicating at least one node is within 3 mm of a segmented blood vessel.

**Fig. 2:**
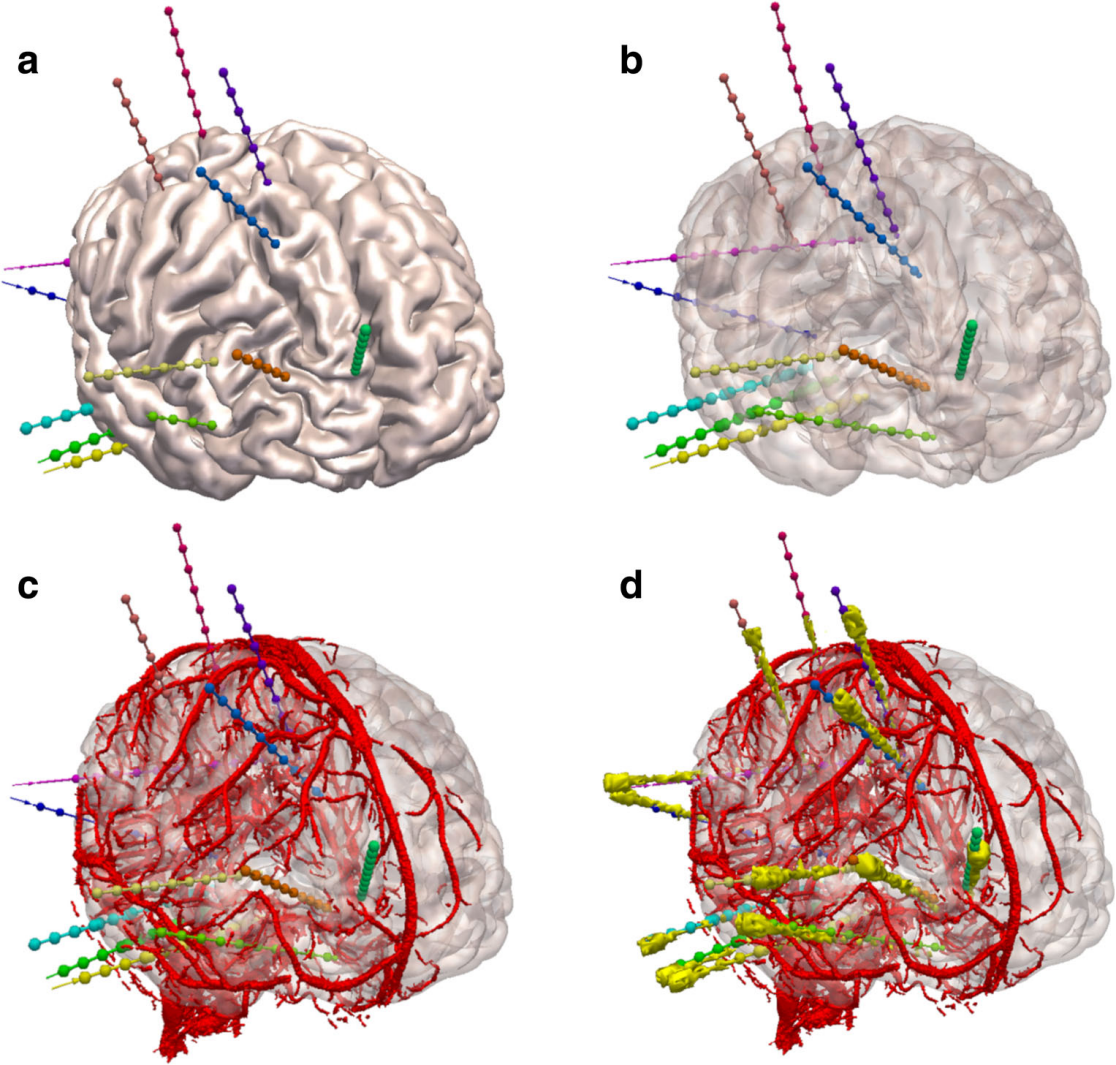
EpiNav generated electrode trajectories: example EpiNav generated implantation from patient 13 with suspected right fronto-temporal onset. **a** Right fronto-lateral view of 3D model of the cortex with the EpiNav generated implantation plan of 13 electrodes. **b** Transparent cortex to allow visualization of the intracerebral course of the planned electrodes. **c** Superimposed vessel segmentation from a right internal carotid artery used for precise planning. **d** Superimposed post-implantation bolt and actual electrode contact segmentation (yellow)

Sulcal models were derived from the whole brain parcellation and were set as no-entry zones to prevent electrodes from passing through the sulci pial boundary where vessels are known to be present (see Fig. 2). The gray matter at the bottom of sulci are sampled by contacts on electrodes passed down the adjacent gyrus. GM was weighted so that trajectories with increased GM sampling were preferentially selected, in order to maximize the efficiency of detecting epileptic activity. Coupled with the sulcal model, this preferentially places electrodes within the gray matter at the depth of sulci. Angle crossing the skull and length restrictions were applied at < 30° from orthogonal and < 90 mm, respectively, based on previous work showing that these parameters generated clinically feasible trajectories when assessed by blinded external experts [13]. Planned trajectories were prevented from being within 10 mm of other trajectories to satisfy local post-SEEG MRI safety guidelines.

Trajectories generated by CAP were labelled as *plan 2* and represent the output of CAP without any human review. Following this *next entry* and *next target* buttons were used to iterate through the CAP planned trajectories in a risk-stratified manner until the most feasible trajectory was found and labelled as *plan 3*. This represents the most feasible trajectory that could be provided with CAP after human review but without precise adjustments. If precise adjustments were required, these were applied and labelled as *plan 4*. The mean risk score of all trajectories in plan 1 was compared to plan 4 and the plan with the lowest risk score was implemented surgically. The plans were then exported to the S7 Stealth station (Medtronic Inc., Minneapolis, MN) for stereotactic implantation. Following implantation, patients underwent both CT and MRI scans within 48 h, which were then coregistered to the generated plans. Any hemorrhage (clinical or asymptomatic) present on the images was reviewed and noted. Repeat CT scans were not routinely performed after removal of the electrodes to prevent unnecessary irradiation unless there was a clinical indication. All other surgical complications were stratified according to the Clavien–Dindo classification [21].

### Statistical Analysis

A prospective sample size calculation revealed that 42 electrode comparisons (~ 5 patients) would be required to detect a 0.1 reduction in risk score assuming a standard deviation (S.D.) of 0.1 and a power 0.9 (*β* = 0.1) and significance level *α* = 0.05, two-tailed. To account for the potential of clustering [22], we required ≥ 118 electrodes (~ 13 patients), assuming a cluster size of 10 electrodes per plan and an intracluster correlation coefficient of 0.2.

Statistical analysis was performed using Stata (Version 15). Comparison of paired trajectory metrics between Plan 1 and 4 was undertaken using mixed-effects linear regression models, with patient-level random effects to account for the clustering of electrodes within patients. Difference estimates together with associated 95% confidence intervals and *p* values for a test of the null hypothesis that the true difference is zero are reported.

Comparison between the different phases of CAP (plans 2–4) was also performed using mixed-effects linear regression models with patient-level random effects to account for the clustering of electrodes within patients. Estimates for each metric are reported for each plan type (with 95% confidence intervals). In each case, a likelihood ratio test was used to obtain a *p* value for tests of the null hypothesis of no difference in the corresponding metric between plans 2 and 4.

## Results

**Fig. 3:**
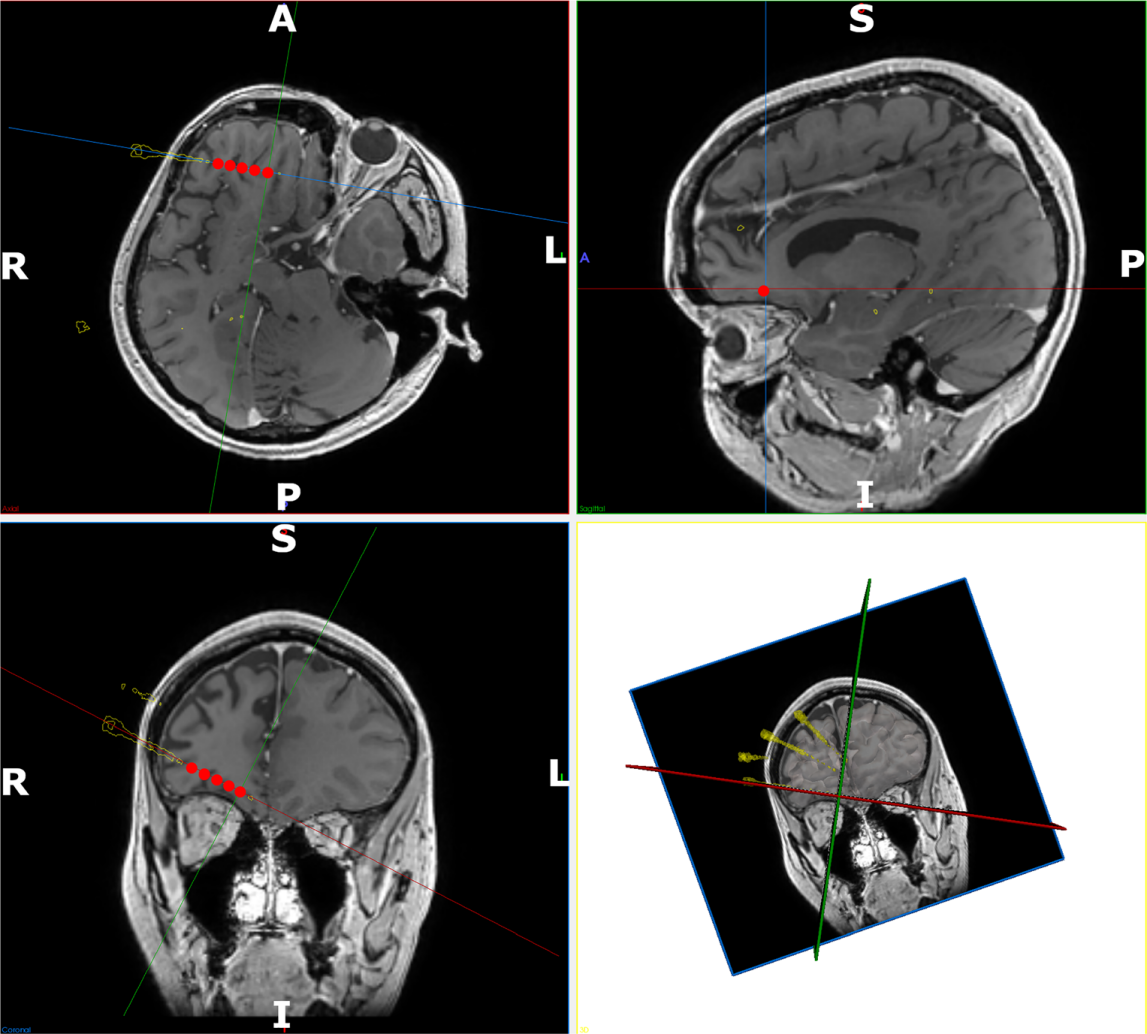
Detailed post-implantation view of active contacts: detailed views of the contacts that were active on the right orbitofrontal electrode at the onset of the seizure. Implemented electrode trajectories segmented from the post-operative CT are shown (yellow) and fused with the preoperative MRI. The electrode contacts active at the onset of the seizure are shown in red. These have been accentuated for clarity. In-line trajectory views (top left and bottom left) as well as probes eye view (top right) and 3D model (bottom right) are shown. Note: the orbitofrontal trajectory passes through the gray matter at the depths of the sulci along the orbitofrontal cortex before terminating in the mesial prefrontal cortex. Electrode conflicts with vessels in the sulcus are averted by preventing the trajectory from crossing sulcal pial boundaries

A total of 125 electrodes (mean of 9.62 electrodes per patient) were implanted (see Table 2), of which 7 were in the left hemisphere (see Figs. 2 and 3 for an example of an implanted CAP generated plan).

**Table 2:** Summary of electrode sampling regions

		Subject number [implanted hemisphere]												
		1 [R]	2 [L]	3 [R]	4 [L]	5 [R]	6 [R]	7 [L]	8 [L]	9 [R]	10 [L]	11 [R]	12 [L]	13 [L]
Temporal	Amygdala	✓	✓	✓	✓		✓	✓	✓	✓	✓	✓		✓
	Anterior hippocampus	✓	✓	✓	✓		✓	✓	✓	✓	✓	✓	✓	✓
	Posterior hippocampus	✓	✓		✓			✓	✓	✓	✓	✓		✓
	Temporo-occipital junction	✓								✓	✓			✓
	Superior temporal gyrus							✓						
	Middle temporal gyrus	✓	✓	✓	✓		✓	✓	✓	✓	✓	✓	✓	✓
Cingulum	Anterior cingulum	✓		✓	✓	✓	✓		✓	✓	✓	✓		✓
	Middle cingulum		✓	✓	✓		✓			✓			✓	
	Posterior cingulum	✓							✓		✓	✓		✓
Frontal	Mesial orbitofrontal cortex	✓	✓	✓	✓	✓	✓		✓	✓	✓	✓	✓	✓
	Lateral orbitofrontal cortex	✓	✓	✓	✓	✓	✓		✓	✓	✓	✓	✓	✓
	Superior frontal gyrus		✓	✓	✓	✓	✓	✓			✓	✓	✓	✓
	Middle frontal gyrus	✓	✓	✓					✓	✓	✓	✓	✓	
	Inferior frontal gyrus			✓	✓	✓			✓	✓	✓	✓	✓	✓
	Mesial prefrontal cortex	✓	✓	✓	✓	✓	✓		✓			✓	✓	✓
	Pre-SMA												✓	
	Anterior SMA		✓			✓	✓						✓	
	Posterior SMA		✓			✓	✓						✓	✓
	Precentral gyrus		✓								✓			
Parietal	Postcentral gyrus		✓			✓								✓
	Superior parietal lobule	✓		✓		✓	✓	✓	✓	✓			✓	✓
	Supramarginal gyrus	✓							✓		✓	✓		✓
	Angular gyrus	✓												
Insula	Anterior Insula	✓	✓	✓	✓	✓		✓	✓	✓	✓			
	Posterior Insula	✓	✓			✓		✓	✓	✓				

Anatomic brain regions sampled by SEEG. Note: the same region may be sampled by more than one electrode and one electrode may sample multiple brain region, e.g. an orbitofrontal electrode implanted using an orthogonal trajectory may enter through the pars orbitalis of the inferior frontal gyrus, sample the lateral orbitofrontal gyrus, mesial orbitofrontal gyrus and terminate in the mesial prefrontal region. Occasionally, the anterior insula may also be sampled using this trajectory

**Fig. 4:**
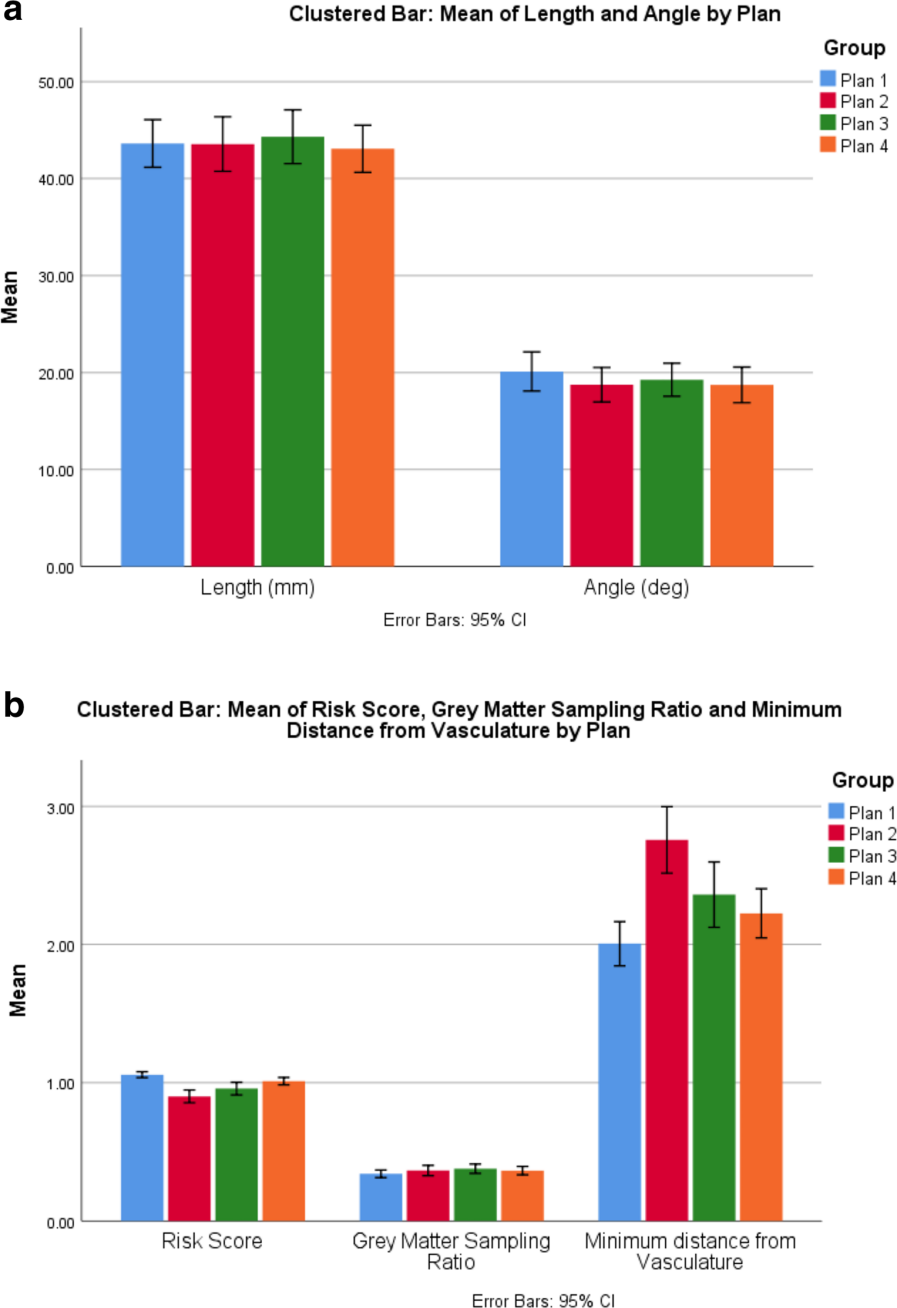
Comparative trajectory metrics between plans: **a** comparison of mean length (mm) and drilling angle to the skull (deg.) and **b** risk score, gray matter sampling ratio, and minimum distance from vasculature (mm) between the different trajectory generation methods (plans 1–4). Error bars represent 95% confidence intervals

At an individual patient level, plans derived following CAP with human review and adjustment (plan 4) had a lower mean risk score compared to the manual plans (plan 1) and were stereotactically implanted in all 13 cases (see Table 3 and Fig. 4). There were no hemorrhages or adverse events following implementation of the plans (125 electrodes) and the target structures were successfully sampled in all cases.

**Table 3:** Metric comparison between manual (plan 1) and final CAP (plan 4)

Metric	Estimate (plan 1–plan 4 difference)	95% confidence interval	*p* value
Length (mm)	0.54	(− 2.94, 4.02)	0.762
Drilling angle (deg.)	1.11	(− 1.88, 4.10)	0.467
GM sampling ratio	−0.02	(− 0.05, 0.00)	0.098
Risk score	0.05	(0.02, 0.08)	0.003
Minimum distance from critical structure (mm)	−0.22	(− 0.43, − 0.01)	0.040

Estimates for differences between plan 1 and plan 4, for each metric, together with associated 95% confidence intervals and *p* values for a test of the null hypothesis that the true difference = 0. The estimates have been obtained from mixed-effects regression models that include within-patients random effects to account for within-patient clustering of electrodes

Group level metrics for the individual plans are shown in Tables 3 and 4. Comparison of trajectory metrics between plans 1 and 4 revealed a significant reduction in risk score (*p* = 0.003) and minimum distance from critical structures (*p* = 0.04), but not intracerebral trajectory length (*p* = 0.76), drilling angle (*p* = 0.47) or gray matter sampling ratio (*p* = 0.10). Subgroup analysis of the trajectory parameters between plans 2 and 4 revealed that risk score and minimum distance following the immediate output of CAP (plan 2) was significantly lower than following the subsequent human interaction (plan 3 and plan 4) *p* = 0.00024 and *p* = 0.001, respectively.

**Table 4:** Metric comparison between different phases of CAP (Plans 2–4)

Metric	Estimate (95% confidence interval)			*p* value*
	Plan 2	Plan 3	Plan 4	
Length (mm)	43.65 (39.39, 47.92)	44.41 (40.15, 48.68)	43.16 (38.90, 47.44)	0.772
Drilling angle (deg.)	18.85 (16.53, 21.18)	19.36 (17.04, 21.68)	18.84 (16.52, 21.16)	0.885
GM sampling ratio	0.37 (0.30, 0.45)	0.39 (0.31, 0.46)	0.37 (0.30, 0.45)	0.704
Risk score	0.90 (0.84, 0.96)	0.96 (0.90, 1.02)	1.01 (0.95, 1.07)	0.00023
Minimum distance from critical structure (mm)	2.76 (2.45, 3.07)	2.36 (2.05, 2.67)	2.23 (1.92, 2.54)	0.001

Estimates for each metric by group from mixed-effects ANOVA models that include patient-level random effects to account for within-patient clustering of electrodes. *p values are shown for likelihood ratio tests of the null hypothesis of no difference in the corresponding outcome variable between plans 2 and 4

**Fig. 5:**
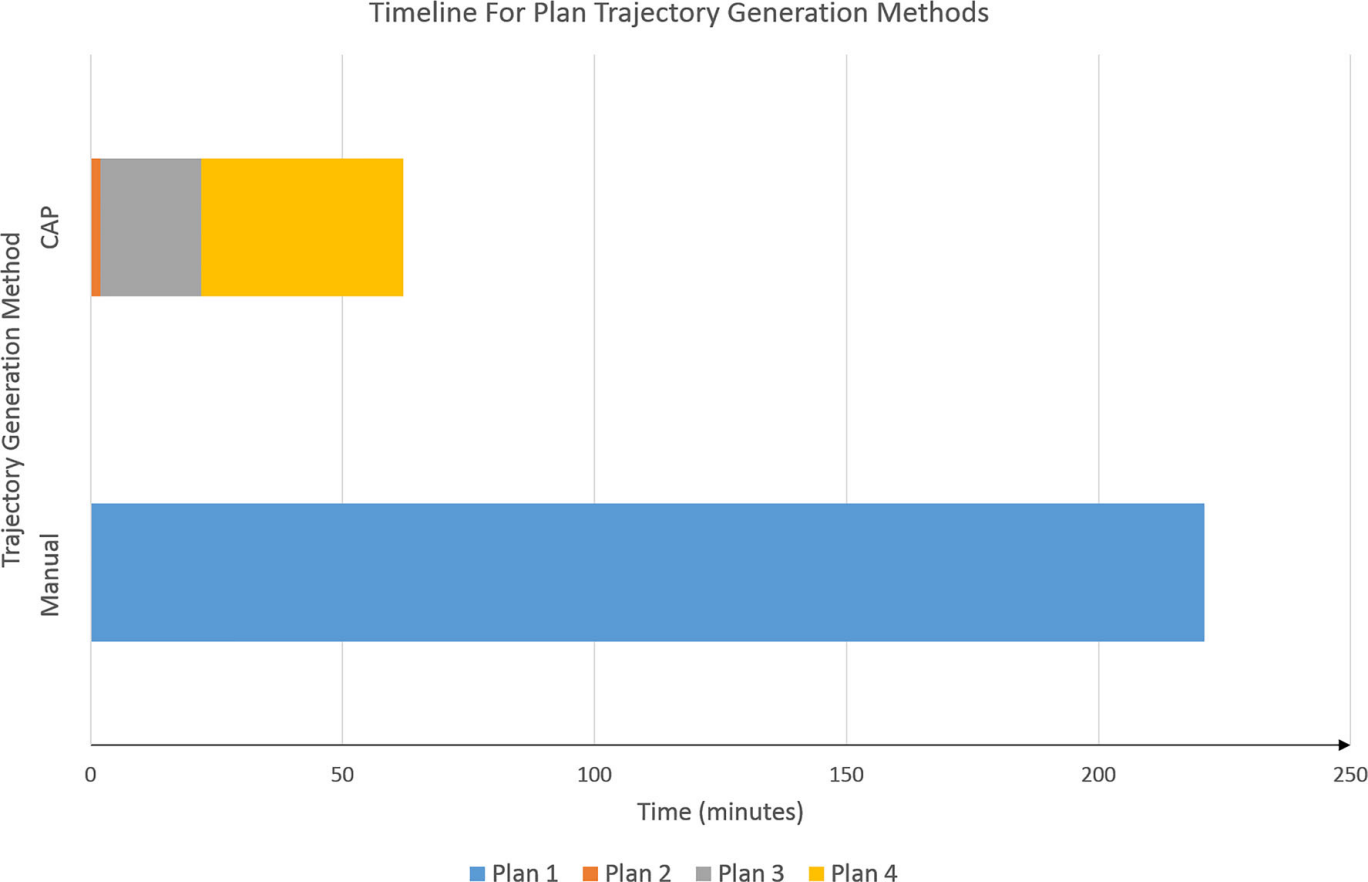
Timeline for SEEG implantation generation for CAP and manually generated trajectories: comparative mean timelines for trajectory generation between CAP and manually planned SEEG implantations. Manual trajectory planning is represented by plan 1. CAP planning consisted of automated trajectory generation (plan 2), followed by semi-automated trajectory alterations by cycling through risk-stratified automated trajectories (plan 3) and manual checking and fine adjustments to the CAP trajectories were required (plan 4). Please note, only plan 4 trajectories were implanted into patients

Computation times for CAP (generation of plan 2) ranged from 34 to 89 s. Review of the trajectories and iteration through the risk-stratified trajectories (plan 3) required an additional 15–20 min. Final precise adjustments (plan 4) and review of all trajectories took an additional 20–40 min depending on the complexity of the implantation (see Fig. 5). Using CAP, the total time for plan generation, individual trajectory review, and precise adjustment took 62 ± 17 min (mean ± S.D.). Manual planning took an average of 221 ± 39 min (mean ± S.D.). Due to the long manual planning duration, this was usually spread over two separate sessions. Overall CAP (plans 2–4) was significantly quicker than manual planning (*p* = 6.4 × 10^−8^).

## Discussion

Before CDSSs can be integrated into the clinical pathway, they must be rigorously tested and externally validated to ensure that they perform optimally across a representative range of patients and institutions (see Table 5 for a summary of the published literature to date along with methodological index for nonrandomized studies (MINORS) [23] scores for study design appraisal) (see supplementary material Table 1 for itemization of MINORS scores).

**Table 5:** Summary of published literature of clinical studies utilizing CAP for SEEG

Publication	CAP platform	MINORS	Type	Number of patients (electrodes)	Parameters optimized	Comments
De Momi et al. (2013)	3D Slicer	16/24	Retrospective	15 (199)	Vessel distance Skull drilling angle Sulci	Single electrode planning Entry and target points manually selected by surgeon and 4.38 mm and 4.27 mm search radius applied respectively No external validation
Zombori (et al 2014)	EpiNav	12/24	Retrospective	6 (30)	Vessel distance Skull drilling angle Electrode length Risk score	Single electrode planning Overall electrode risk score, length, and drilling angle were improved with CAP
De Momi et al. (2014)	3D Slicer	16/24	Retrospective	3 (24)	Vessel distance Skull drilling angle Adherence to planned entry and target structure Cortex curvature value	Multielectrode planning 1.6 mm safety margin from vasculature within 2.5 cm of skull entry point and 1 mm safety margin thereafter Maximum drilling angle 40° Minimum distance from vessel was significantly improved with multielectrode planning No external validation
Zelmann et al. (2014)	MINC toolkit	14/24	Retrospective	6 (27)	Vessel distance Sulci Ventricles Gray matter sampling Target volume sampling	Multielectrode planning Only amygdala and hippocampus targeted Automated trajectories improved target volume sampling, distance from vasculature and gray matter contact 25/27 trajectories were rated feasible
Zelmann et al. (2015)	MINC toolkit	14/24	Retrospective	20 (116)	Risk score ROI recording volume Gray matter sampling Skull drilling angle	Multielectrode planning Only 3 electrodes (amygdala, anterior hippocampus, and posterior hippocampus) planned with target structures defined as ROIs. Single neurosurgeon did feasibility assessment on all patients. A second neurosurgeon scored 12 patients. No external validation Automated trajectories were statistically safer overall and rated more feasible than those that were manually planned. Insertion angle was higher with automated trajectories
Nowell et al. (2016)	EpiNav	16/24	Retrospective	18 (166)	Electrode length Skull drilling angle Risk score Vessel distance Gray matter sampling Sulci	Multielectrode planning 3 mm safety margin from vasculature along entire length of trajectory with risk profile graphic Surgeon manually selects target point Able to generate 98.2% of the required trajectories External blinded evaluation revealed 79% were feasible for implantation without further adjustment
Scorza et al. (2017)	3D Slicer	14/24	Retrospective	20 (253)	Vessel distance Sulcal avoidance Skull drilling angle Electrode conflicts	Multielectrode planning 4 mm safety margin from vasculature within 1 cm of skull entry point and 1 mm safety margin thereafter Entry and target points manually selected by surgeon and 7 mm and 3 mm search radius applied respectively Improvement in optimization parameters in 98% of electrodes. No feasibility ratings of trajectories or external validation undertaken.
Sparks et al. (2017)a	EpiNav	16/24	Retrospective	18 (165)	Electrode length Skull drilling angle Risk score Vessel distance Gray matter sampling Sulci	Multielectrode planning 3 mm safety margin from vasculature along entire length of trajectory with risk profile graphic Surgeon manually selects target point Entry structure risk map generation Improvement in risk, gray matter sampling, intracerebral length, and drilling angle with CAP Skull template to remove infeasible entry points
Sparks et al. (2017)b	EpiNav	16/24	Retrospective	20 (190)	Electrode length Skull drilling angle Risk score Vessel distance Gray matter sampling Sulci	Multielectrode planning 3 mm safety margin from vasculature along entire length of trajectory with risk profile graphic Entry and target regions defined as anatomic ROIs allowing algorithm to define optimal entry and target points Entry and target structure risk map generation Iterative relaxation of hard constraints if suitable trajectories cannot be found External blinded feasibility ratings were 97% for manual and 90% for CAP generated trajectories
Vakharia et al. (2017)	EpiNav	20/24	Retrospective	13 (116)	Electrode length Skull drilling angle Risk score Vessel distance Gray matter sampling Sulci	Multielectrode planning 3 mm safety margin from vasculature along entire length of trajectory with risk profile graphic Entry and target regions defined as anatomic ROIs allowing algorithm to define optimal entry and target points External review of manual and CAP trajectories in blinded fashion revealed no difference in feasibility Improvement in risk, gray matter sampling, intracerebral length and drilling angle with CAP
Vakharia et al. (2018)*	EpiNav	24/24	Prospective	13 (125)	Electrode length Skull drilling angle Risk score Vessel distance Gray matter sampling Sulci	Multielectrode planning First prospective CAP study in which CAP trajectories were implemented with no adverse events Significant improvement in risk score

*Current study

Over the last 5 years, CAP algorithms have significantly advanced. Initial studies implemented many of the single trajectory planning features [24, 25] that had been developed previously for deep brain stimulation [26]. As SEEG schema contains many more electrodes and the target points are anatomically more varied, multitrajectory planning was required [10, 12, 27]. This added an additional level of complexity to CAP planning as not only did individual electrodes need to be optimized with regard to planning parameters, but they could not come within a user-defined distance of each other. Various parameters for minimum vascular distance, sulcal avoidance, risk calculation, and drilling angle through the skull have been implemented and these studies have shown significant improvements in planning time. With the addition of patient-specific whole brain parcellations, entry and target structures no longer need to be manually selected but whole brain anatomical regions can now be specified. This helps to further automate the process and increase the potential number and safety of generated trajectories [13, 14]. To date, all previous studies have been retrospective comparisons in which previous manually planned and implemented trajectories were replanned utilizing CAP and metrics compared back to the manual plans. Due to the low incidence of intracranial hemorrhage associated with SEEG, most CAP studies have adopted a surrogate metric in the form of a risk score [12, 26, 28–30], which is the cumulative distance from the vasculature, for comparison. To validate the clinical feasibility of the trajectories, these were rated by expert neurosurgeons [10, 11, 14, 31].

We have previously undertaken a retrospective clinical assessment of SEEG planning with EpiNav [13]. Consecutive patients that had undergone manual planning and electrode implantation were selected from a prospectively maintained database and the implantation schema was replanned using CAP. The resulting trajectory metrics revealed that CAP trajectories significantly improved trajectory length, drilling angle, gray matter sampling ratio, risk, and minimum distance from vasculature. The trajectories were also externally validated by five expert neurosurgeons that were blinded to the trajectory generation method. There was no significant difference in feasibility between manual and CAP generated electrodes. The implication of this was that CAP could generate SEEG trajectories that are potentially safer and more efficient than those planned manually in a fraction of the time. This study also instituted a sulcal model that prevents electrodes from crossing the sulcal pial boundaries in order to reflect the current surgical practice at our institution. We acknowledge that there is variability in surgical practice with regard to crossing sulcal pial boundaries and accordingly this constraint can be turned on or off at the surgeon’s discretion. The intent of the sulcal model is to prevent trajectories passing through sulcal pia where vasculature is known to reside (see Fig. 2). Our practice to sample gray matter at the bottom of sulci, which is a common site for focal cortical dysplasia, is to direct trajectories obliquely through the adjacent gyrus. The preferential GM sampling feature facilitates efficient sampling of all selected gray matter targets. The current prospective study builds upon our retrospective experience with CAP for SEEG, validating this as a CDSS for trajectory planning.

During the generation of CAP trajectories, we assessed metrics at each stage to replicate the expected “real-world” clinical application. As a CDSS, it is intended that the recommended output of CAP (plan 2) be reviewed by the operating neurosurgeon and any potential modifications be made by iterating through the CAP trajectories in a risk-stratified manner (plan 3). This allows the neurosurgeon to customize trajectories to fit their usual practice while also utilizing CAP to ensure that the trajectory carries the lowest risk. If required, further modifications can be made by the neurosurgeon setting precise entry and target points (plan 4) prior to implantation. For the purpose of this prospective validation study, we compared the manual plan (plan 1), made in advance of CAP, with the final CAP-assisted plan ready for implantation (plan 4). In all cases, plans carrying the lowest mean risk score were stereotactically implanted. No patients had an adverse event related to the planning or implantation of the CAP generated trajectories. Unlike in previous studies [11, 13], there was no significant difference in intracerebral length, drilling angle to the skull, or GM sampling ratio between manual and implemented CAP trajectories. This is most likely due to the evolving nature in which manual planning was performed, whereby the preoperative 3D models generated for use with CAP were also available to the neurosurgeon.

It should be noted that there is a significant distinction between computer-assisted and computer-autonomous planning. The former requires an expert neurosurgeon to review and modify the suggested plans as necessary prior to stereotactic implantation. Due to the complexity of SEEG planning and variability in implantation methods as well as surgeon planning practices, it is unlikely that computer-autonomous algorithms will be available in the near future. We have previously found that manually planned and implemented trajectories rated by external blinded neurosurgeons were deemed feasible in only 70%. In each of these cases, the manually planned trajectories were by definition feasible as they had already been implanted without complication. This highlights the lack of consensus between stereotactic neurosurgeons regarding planning parameters and is likely to be a hurdle to widespread adoption of computer-autonomous planning. Furthermore, the variability in acquisition parameters for preoperative MRI and methods for vascular imaging mean the results of CAP will vary between institutions. In this study, we employ DSCA to provide the greatest segmentation of intracranial vasculature, although not all institutions acquire this [32]. It still remains unclear what is the critical vessel size for visualization for safe SEEG. The ability for the neurosurgeon to be able to modify CAP output is key to customizing trajectories based on individual surgeon preferences and building user confidence in the algorithms.

There are limitations to this study. It would have been methodologically superior to perform a prospective, randomized controlled trial of CAP *versus* manual trajectory planning. As there have not been any prospective studies of CAP to date, we decided it would be safer to independently generate CAP and manual plans for comparison and implant those with the lowest risk score. As the position of individual trajectories impacts upon other trajectories in the plan, we compared the mean risk score for the overall plan and not at an individual electrode level. Furthermore, all patients and implantations were performed at a single institution where uniform imaging protocols were performed on all patients. It is unclear whether the same results would be achieved by other institutions employing different imaging strategies. We have suggested parameters for suitable image acquisition protocols using different MRI scanners (supplementary material Table 2) so this can be replicated at other centers. We acknowledge the small sample size of the study (*n* = 125 electrodes in 13 patients) but emphasize that even when controlling for clustering within patients the study was well powered to detect the study primary end-point (power = 0.9 to a difference in risk score of ≥ 0.1). A further limitation of this study, and one that is ubiquitous in all CAP algorithms, is the reliance on a risk score [25, 26, 28, 29]. Due to the low incidence of hemorrhage from SEEG, a prohibitively large sample size would be required to undertake a study in which reduction in hemorrhage rate was the primary outcome. Given that hemorrhage must occur from conflict with a blood vessel (visualized or not by modern imaging techniques) and that exploitation of avascular channels during trajectory planning is the primary goal of the surgeon, we apply the pragmatic tenet that hemorrhage is less likely to occur the further an electrode is placed from an intracranial vessel. The risk score is, therefore, an objective means of quantifying the size of the avascular corridor. Future studies should aim to be multicenter in nature to assess the external robustness of the algorithm and feasibility in the hands of different neurosurgeons. It would also be methodologically optimal if the neurosurgeon was blinded to the generation method but still retained the ability to modify the plans prior to implantation. In reality, surgeon blinding is difficult to implement as the surgeon performing the implantation would have to be different to the surgeon performing the manual planning.

Currently, EpiNav supports the direct export of CAP plans to the S7 Stealth Station (Medtronic Inc.) for implantation. Future developments may include export formats that are compatible with other devices, e.g. Leksell frame. We will also aim to improve the feasibility of the immediate CAP output (plan 2) and reducing the modifications required by the surgeon (plans 3 and 4). Given the significant variability in surgeons’ preference for trajectory planning, this will require customization of CAP to the individual surgeon’s practice. To this end, we propose the generation of spatial priors for specific trajectories that will define commonly used entry and target zones.

## Conclusion

CAP provides clinically feasible SEEG trajectory plans with improved safety metrics in one third of the time required for manual planning. Incorporating automated SEEG planning into the clinical workflow is possible with the use of EpiNav as a CDSS. We have itemized each stage of the trajectory generation pathway and highlighted the ability of the surgeon to modify the trajectories based on their individual planning preferences in a risk-stratified manner. When the final CAP trajectories were directly compared with manual plans, they returned lower mean risk scores in all cases and were prospectively stereotactically implanted without complication. EpiNav is a significant advance in the planning of SEEG trajectories and has application for other stereotactic neurosurgical procedures including planning cranial laser interstitial thermal therapy (LITT), deep brain stimulation, focal therapy delivery, brain biopsies, and shunt catheter placement.

## Electronic Supplementary Material

ESM 1 (DOCX 14 kb)

ESM 2 (DOCX 31 kb)

ESM 3 (DOCX 57 kb)
