# Supplementary material for: Computer-Assisted Planning for Stereoelectroencephalography (SEEG)
Source: Neurotherapeutics. 2019 Aug 20;16(4):1183–97. doi: 10.1007/s13311-019-00774-9 (PMC6985077; doi:10.1007/s13311-019-00774-9)
Supplement: Supplementary file 3 — (DOCX 57 kb) [file 13311_2019_774_MOESM3_ESM.docx]

Supplementary table 1: Derivation of MINORS scores[32]

| Publication | Aims clearly stated | Consecutive patients | Prospective data collection | Appropriate endpoints | Blinded evaluation | Appropriate follow-up period | Loss to follow up <5% | Prospective power calculation | Adequate control group | Prospective control | Baseline equivalence | Adequate statistics | Total |
| --- | --- | --- | --- | --- | --- | --- | --- | --- | --- | --- | --- | --- | --- |
| De momi et al (2013) | 2 | 0 | 0 | 2 | 2 | 2 | 2 | 0 | 2 | 0 | 2 | 2 | 16/24 |
| De momi et al (2014) | 2 | 0 | 0 | 2 | 0 | 2 | 2 | 0 | 2 | 0 | 2 | 2 | 14/24 |
| Zombori et al (2014) | 2 | 0 | 0 | 2 | 2 | 2 | 2 | 0 | 2 | 0 | 2 | 2 | 16/24 |
| Zelmann et al (2014) | 2 | 0 | 0 | 2 | 0 | 2 | 2 | 0 | 2 | 0 | 2 | 2 | 14/24 |
| Zelmann et al (2015) | 2 | 0 | 0 | 2 | 0 | 2 | 2 | 0 | 2 | 0 | 2 | 2 | 14/24 |
| Nowell et al (2016) | 2 | 0 | 0 | 2 | 2 | 2 | 2 | 0 | 2 | 0 | 2 | 2 | 16/24 |
| Scorza et al (2017) | 2 | 0 | 0 | 2 | 0 | 2 | 2 | 0 | 2 | 0 | 2 | 2 | 14/24 |
| Sparks et al (2017)a | 2 | 0 | 0 | 2 | 2 | 2 | 2 | 0 | 2 | 0 | 2 | 2 | 16/24 |
| Sparks et al (2017)b | 2 | 0 | 0 | 2 | 2 | 2 | 2 | 0 | 2 | 0 | 2 | 2 | 16/24 |
| Vakharia et al (2017) | 2 | 2 | 0 | 2 | 2 | 2 | 2 | 2 | 2 | 0 | 2 | 2 | 20/24 |
| Vakharia et al - (2018) * | 2 | 2 | 2 | 2 | 2 | 2 | 2 | 2 | 2 | 2 | 2 | 2 | 24/24 |

* Current Study

Supplementary table 2: Suggested MRI scanner parameters for CAP planning

a) 3T Siemens Prisma (software version VE11C)

| Sequence | 3D T1 MPRAGE (Acquisition time: 5m19s)  Inversion-prepared gradient echo | MR Venography (Acquisition time: 16m05s)  3D Phase-contrast | MR Angiography (Acquisition time: 9m03s)  3D Phase contrast |
| --- | --- | --- | --- |
| Geometry | Orientation: Coronal  In-plane FOV of 25.6x25.6 cm with an acquisition matrix 256x256 (GRAPPA factor 2)  240 slice locations of 1.0 mm thickness  Reconstructed voxel size: 1.0x1.0x1.0 mm | Orientation: Axial  In-plane FOV of 22x22 cm with an acquisition matrix 512x512 (GRAPPA factor 3)  Phase FOV: 75%  Phase-encoding direction R>>L  208 slice locations of 0.6 mm thickness with slice resolution 50%  Phase partial Fourier: off  Reconstructed voxel size: 0.43x0.43x0.60 mm | Orientation: Axial  In-plane FOV of 22x22 cm with an acquisition matrix 512x512 (GRAPPA factor 3)  Phase FOV: 75%  Phase-encoding direction R>>L  208 slice locations of 0.6 mm thickness with slice resolution 50%  Phase partial Fourier: off  Reconstructed voxel size: 0.43x0.43x0.60 mm |
| Sequence details | TR/TE/TI = 7.4 / 2.7 / 909 ms  Segment time (time between inversion pulses): 2300 ms  Flip angle: 8 degrees  BW: 200 Hz/pix | TR/TE = 76.55 / 8.31 ms  Flip angle: 8 degrees  BW: 425 Hz/pix | TR/TE = 46.45 / 6.76 ms  Flip angle: 15 degrees  BW: 425 Hz/pix |
| Vascular |  | Velocity encoding: 15 cm/s | Velocity encoding: 80 cm/s |
| Other | Distortion Corr. (3D)  Pre-scan Normalize | Suppression slab inferior to FOV |  |

b) 3T GE MR750 (software version DV24_R02)

| Sequence | 3D T1 MPRAGE (Acq time: 4m17s)  Scan type: 3D Gradient Echo Fast SPGR  Imaging Options: IR Prepared, ASSET, EDR | MR Venography (Acq time: 13m7s)  Scan type: 3D Vascular Inhance Velocity  Imaging Options: ASSET, ZIP2 | MR Angiography (Acq time: 5m49s)  Scan type: 3D Vascular Inhance Velocity  Imaging Options: ASSET, ZIP2 |
| --- | --- | --- | --- |
| Geometry | Orientation: Coronal  In-plane FOV of 25.6x25.6 cm with an acquisition matrix 256x256 (ASSET acceleration 2)  Phase FOV 0.8  224 slice locations of 1.0 mm thickness  Reconstructed voxel size: 1.0x1.0x1.0 mm | Orientation: Axial  In-plane FOV of 22x22 cm with an acquisition matrix 384x256 (ASSET acceleration 2)  Phase FOV 0.9  124 slice locations of 1.2 mm thickness  Reconstructed voxel size: 0.43x0.43x0.60 mm | Orientation: Axial  In-plane FOV of 22x22 cm with an acquisition matrix 384x256 (ASSET acceleration 2)  Phase FOV 0.9  124 slice locations of 1.2 mm thickness  Reconstructed voxel size: 0.43x0.43x0.60 mm |
| Sequence details | TR/TE/TI = 7.4 ms / Min Full / 400 ms  Flip angle: 11 degrees  BW: 31.25 | TR/TE = Minimum  Flip angle: 8 degrees  BW: 31.25 | TR/TE = Minimum  Flip angle: 8 degrees  BW: 31.25 |
| Vascular |  | Flow analysis off  Velocity encoding: 15 cm/s  Flow recon type complex difference  Acquisition flow direction images: all add flow images, magnitude | Flow analysis off  Velocity encoding: 80 cm/s  Flow recon type complex difference  Acquisition flow direction images: all add flow images, magnitude |
| Other | 3D Geometry correction: on  Intensity inhomogeneity correction: PURE | 3D Geometry correction: on  Intensity inhomogeneity: PURE  Advanced: 80% pfkr  Suppression slab inferior to FOV  Chemical fat saturation | 3D Geometry correction: on  Intensity inhomogeneity: PURE  Advanced: 80% pfkr |

c) 3T Philips Achieva

| Sequence | 3D T1 MPRAGE (Acq time: 6m32s)  Inversion-recovery 3D TFE | MR Venography (Acq time: 8m58s)  3D T1 Fast Field echo, phase contrast | MR Angiography (Acq time: 5m30s)  3D T1 Fast Field echo, phase contrast |
| --- | --- | --- | --- |
| Geometry | Orientation: Sagittal  In-plane FOV of 25.6x25.6 cm with an acquisition matrix 256x256 (SENSE factor 2)180 slice locations of 1.0 mm thickness  Reconstructed voxel size: 1.0x1.0x1.0 mm | Orientation: Axial  In-plane FOV of 23x18 cm with an acquisition matrix 384x212 (SENSE factors: RL=3, IS=2)  Scan percentage: 70.87%  248 slice locations of 1.2 mm thickness (overcontiguous slices)  Reconstructed voxel size: 0.45x0.45x0.60 mm | Orientation: Axial  In-plane FOV of 23x18 cm with an acquisition matrix 384x212 (SENSE factors: RL=3, IS=2)  Scan percentage: 70.87%  248 slice locations of 1.2 mm thickness (overcontiguous slices)  Reconstructed voxel size: 0.45x0.45x0.60 mm |
| Sequence details | TR/TE/TI = 6.9 / 3.1/ 867 ms  Segment time (time between inversion pulses): 3000 ms  Flip angle: 8 degrees | TR/TE (both set to shortest) = 21 / 8.3 ms  Flip angle: 8 degrees  Halfscan: yes  water-fat shift: maximum  BW: 114.6 Hz | TR/TE (both set to shortest) = 13 / 6.7 ms  Flip angle: 8 degrees  Halfscan: yes  water-fat shift: maximum  BW: 114.6 Hz |
| Vascular |  | Velocity encoding: 15 cm/s | Velocity encoding: 80 cm/s |
| Other | 3D Geometry correction: on | fold-over direction: RL  fat shift direction: P  NSA: 2  REST slab: type=parallel, thickness=60 mm, position=feet, gap=default, power=1 | fold-over direction: RL  fat shift direction: P  NSA: 2 |

Note: Parameters may have to be altered on different models.
