# Supplementary material for: Computer-Assisted Planning for Stereoelectroencephalography (SEEG)
Source: Neurotherapeutics. 2019 Aug 20;16(4):1183–97. doi: 10.1007/s13311-019-00774-9 (PMC6985077; doi:10.1007/s13311-019-00774-9)
Supplement: Supplementary file 1 — (DOCX 14 kb) [file 13311_2019_774_MOESM1_ESM.docx]

### Dear Professor Mouradian,

Refractory epilepsy is a common and devastating illness and can be cured by neurosurgery if the source of epilepsy in the brain can be pinpointed and removed. In many patients, identifying the epileptic focus requires the insertion of multiple (8-16) stereotactically placed intracerebral electrodes.

The placement of these electrodes carries risk of causing cerebral haemorrhage and deficits, and precise planning of trajectories is necessary to minimise the risk of causing damage and to have a good chance of identifying the epileptic focus.

Traditionally, electrode trajectories have been planned manually, which is very laborious and time-consuming for an expert multidisciplinary team.

We have pursued epilepsy surgery and image-guidance of surgery for many years, ^1-3^ with a succession of Wellcome Trust Programme grants.

The current paper is the result of a prospective comparison of conventional manual planning of multiple electrode trajectories and a computer-assisted clinical decision support software that we have created. This is the first prospective clinical validation study comparing such software with manual planning for stereotactic neurosurgery.

In all cases, trajectories derived from computer-assisted planning provided the lowest risk score, with a very significant time-saving, and were implanted into patients without adverse sequelae. We conclude that computer-assisted electrode trajectory planning is a significant step forward and the technology can be readily applied more widely: for directing trajectories for biopsies, shunts, deep brain simulation, lesion creation and local drug delivery.

Thank you for considering this original research article entitled ‘Computer Assisted Planning For Stereoelectroencephalography (SEEG)’ for publication in Neurotherapeutics. We feel this is an important study that marks the integration of software support into clinical neurosurgical practice and will be of interest to a broad readership.

Yours Sincerely,

Dr Vejay N. Vakharia MRCS (Corresponding author), Prof. John S. Duncan FRCP and Prof. Sebastien Ourselin PhD, on behalf of all authors.

**References**

1. De Tisi J, Bell GS, Peacock JL, McEvoy Aw, Harkness WFJ, Sander JW, Duncan JS. The long-term outcome of adult epilepsy surgery, patterns of seizure remission and relapse: a cohort study. Lancet. 2011;378(9800):1388-95.
2. Duncan JS, Winston GP, Koepp MJ, Ourselin S. Brain imaging in the assessment for epilepsy surgery. Lancet Neurology 2016 Apr;15(4):420-33.
3. Vakharia VN, Duncan JS, Witt JA, Elger CE, Staba R, Engel J. Getting the best outcomes from epilepsy surgery. Ann Neurol. 2018 Apr;83(4):676-690.
